# Animal Bite Injuries in Children: Review of Literature and Case Series

**DOI:** 10.5005/jp-journals-10005-1410

**Published:** 2017-02-27

**Authors:** Aviral Agrawal, Pradeep Kumar, Ruchi Singhal, Virendra Singh, Amrish Bhagol

**Affiliations:** 1 Senior Resident, Department of Oral and Maxillofacial Surgery, Kalpana Chawla Government Medical College, Karnal, Haryana, India; 2 Dental Surgeon, Department of Oral and Maxillofacial Surgery, Postgraduate Institute of Dental Sciences, Rohtak, Haryana, India; 3 Senior Resident, Department of Pedodontics, Postgraduate Institute of Dental Sciences, Rohtak, Haryana, India; 4 Professor and Head, Department of Oral and Maxillofacial Surgery, Postgraduate Institute of Dental Sciences, Rohtak, Haryana, India; 5 Assistant Professor,Department of Oral and Maxillofacial Surgery, Postgraduate Institute of Dental Sciences, Rohtak, Haryana, India

**Keywords:** Animal bite injuries, Dog bites, Facial trauma.

## Abstract

**Introduction:**

Maxillofacial region in children is particularly vulnerable to animal bite injuries. These injuries may range from insignificant scratches to life-threatening neck and facial injuries. Children are the common victims, particularly of dog bites.

**Materials and methods:**

Three cases of animal bite injuries in children with their clinical presentation and their management are being presented along with review of literature. Surgical management included cleansing and primary closure of the wound. Rabies and tetanus prophylaxis were given.

**Discussion:**

The most common site of injury was the face. For the facial injuries, the most frequently affected area was the middle third (55%), also called as the “central target area.” The small stature of children, the disproportionate size of the head relative to the body, their willingness to bring their faces close to the animal, and limited motor skills to provide defense are believed to account for this. The resulting soft-tissue injuries can vary in relation to their extent. Treatment involved initial surgical exploration, and secondary repair later depending on the severity of the injury.

**Conclusion:**

Prompt assessment and treatment can prevent most bite wound complications. Early management of such injuries usually guarantees satisfactory outcome. Prevention strategies include close supervision of child-dog interactions, better reporting of bites, etc.

**How to cite this article:**

Agrawal A, Kumar P, Singhal R, Singh V, Bhagol A. Animal Bite Injuries in Children: Review of Literature and Case Series. Int J Clin Pediatr Dent 2017;10(1):67-72.

## INTRODUCTION

Facial trauma in children represents a significant medical and public health issue.^1-4^ A considerable proportion of skeletal and soft-tissue injuries of the face results from animal bite injuries, mostly due to dog bites.^5^ In the UK, it is estimated that dog attack injuries are responsible for an average of 250,000 minor injuries and emergency unit attendances each year,^6^ and in the USA, an average of 4.7 million dog bites occur each year^7^; many bites probably go unreported. Children, in particular, are more likely to experience dog bite injuries compared with adults, with children aged between 5 and 9 years considered to be the most at risk.^6,8^ Being the most exposed part of the body, the face is particularly vulnerable to such injuries.^9-12^ Among the victims of dog attacks, most studies showed a male preponderance.^10,13,14^ The types of wounds encountered range from insignificant scratches to life-threatening neck and facial injuries. The tissue defects may be superficial, but they can even cause amputations, including severe vascular and nerve or bony destruction. We present three cases of dog bite attacks in young children and their management.

## CASE REPORTS

### Case 1

**Fig. 1:**
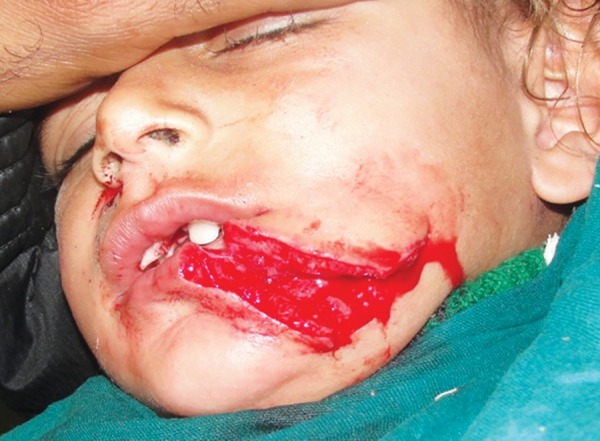
Deep lacerated wound in a 3-year-old girl

**Fig. 2:**
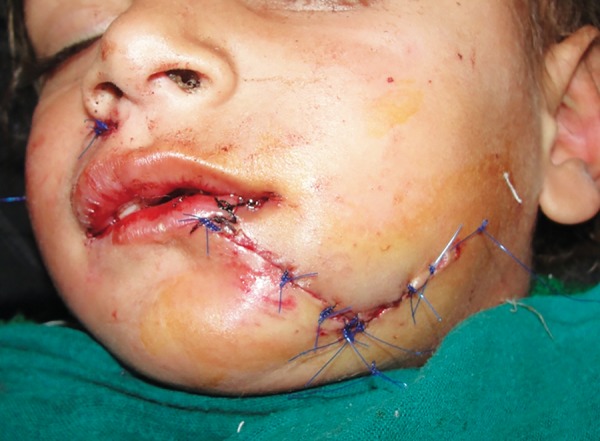
Sutured lacerated wound

**Fig. 3:**
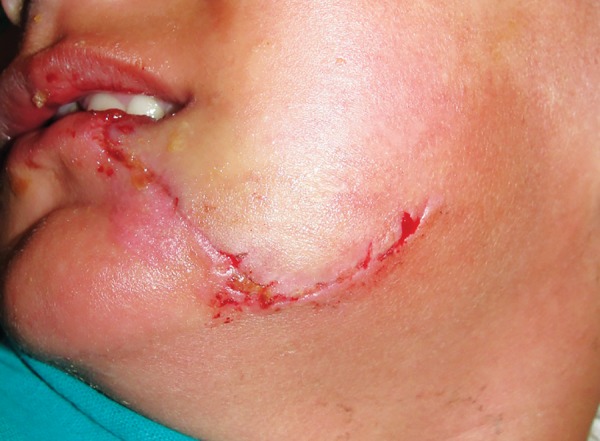
Follow-up picture after removal of sutures

A 3-year-old girl reported to the emergency department following an attack by a stray dog. She was otherwise fit and well, and had no relevant medical history or known allergies. A deep laceration wound was present extending from the left side of lower lip to the lower border of mandible (Fig. 1). A small laceration was present on the right nasolabial fold. Intraoral examination revealed that maxillary deciduous central incisors were slightly extruded and mobile. Her soft-tissue wounds were thoroughly debrided and irrigated with normal saline and hydrogen peroxide. The laceration was sutured with 4-0 round body vicryl and 4-0 reverse cutting prolene suture material (Fig. 2). The luxated maxillary incisors were stabilized with composite splinting. The parents were informed about the postoperative wound management. Tetanus and rabies prophylaxis were evaluated. The child was reviewed after 1 week and sutures were removed (Fig. 3). The patient was kept on regular follow-up for 3 months.

**Fig. 4:**
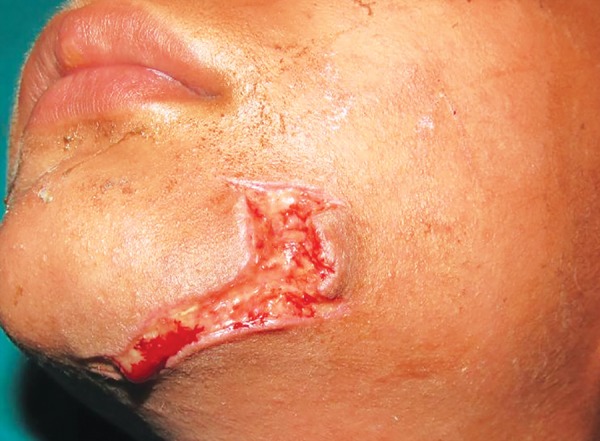
Lacerated wound in a 13-year-old boy

**Fig. 5:**
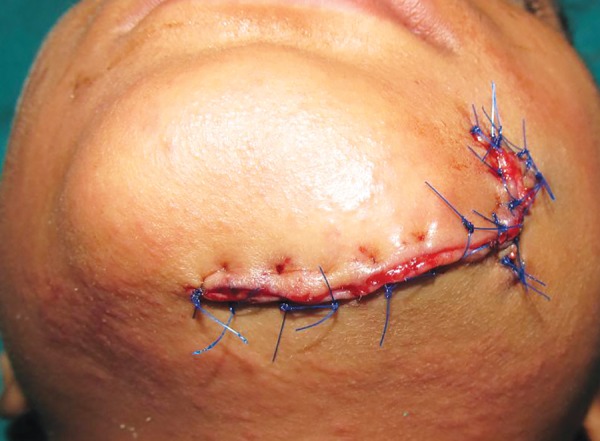
Sutured lacerated wound with vicryl and prolene

### Case 2

**Fig. 6:**
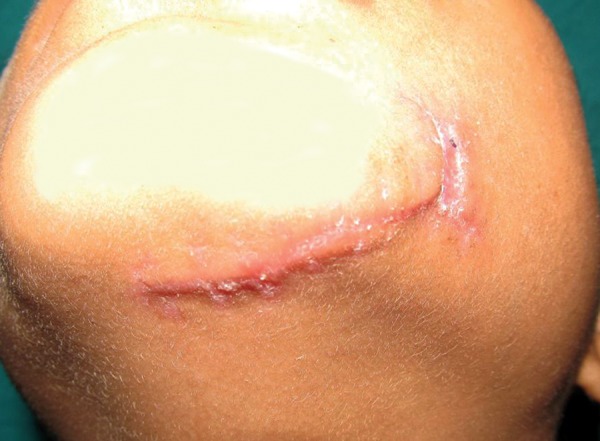
Follow-up picture showing healed wound

A 13-year-old boy reported to the emergency department following an attack by a stray dog. He was otherwise fit and well and had no relevant medical history or known allergies. A deep laceration wound was present on the left side of face extending 1 cm below and lateral to lower lip up to the lower border of mandible in the midline of face (Fig. 4). His soft-tissue wounds were thoroughly debrided and irrigated with normal saline and hydrogen peroxide. The laceration was sutured with vicryl and prolene suture material (Fig. 5). The parents were informed about the postoperative wound management. Tetanus and rabies prophylaxis were evaluated. The child was reviewed after 1 week and sutures were removed (Fig. 6). The patient was kept on regular follow-up for 3 months.

### Case 3

**Fig. 7:**
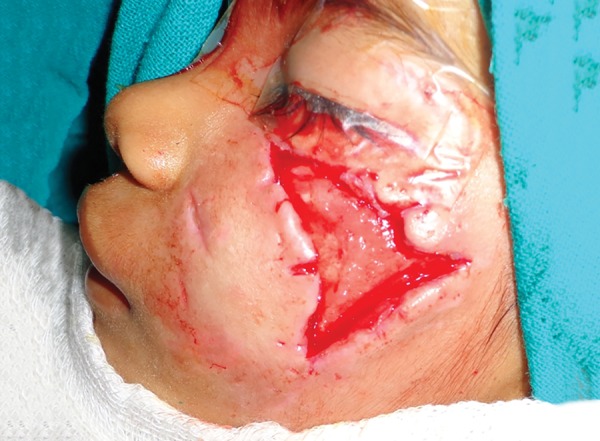
Lacerated wound in a 6-year-old girl

**Fig. 8:**
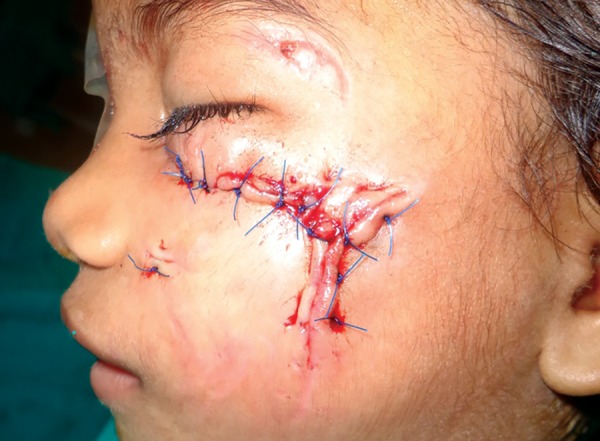
Sutured lacerated wound with vicryl and prolene material

A 6-year-old girl reported to the department with infected suture wound. Parents gave history of an attack by a stray dog, which was sutured by some private practitioner. The wound showed sign of infection with pus collection. She was otherwise fit and well and had no relevant medical history or known allergies. An infected laceration wound was present below the left eye extending up to the middle of the cheek. The sutures were removed and the margins of wound were refreshed with surgical blade (Fig. 7). Her soft-tissue wounds were thoroughly debrided and irrigated with normal saline and hydrogen peroxide. The laceration was sutured with vicryl and prolene suture material (Fig. 8). The parents were informed about the postoperative wound management. Tetanus and rabies prophylaxis were evaluated. The child was reviewed after 1 week and sutures were removed. The patient was kept on regular follow-up for 3 months.

## DISCUSSION

Animal bites have been a major public health problem. Children are the most common victims, particularly of dog bites.^15^ The most common site of injury was the face.^9-12^ For the facial injuries, the most frequently affected area was the middle-third (55%).^13^ This reflects the findings of Palmer and Rees who called this the “central target area.”^16^ The small stature of children, the disproportionate size of the head relative to the body, their willingness to bring their faces close to the animal, and limited motor skills to provide defense are believed to account for this.^4,17^

A study showed that the risk factors for dog attacks include school-aged children (but highest rate of serious injury from dog bite is in children under 5 years of age),^18^ male, households with dogs, certain breeds (German shepherds, bull terriers, blue/red heelers, dobermans, and rottweilers), and male dogs. Most of the cases involve a known dog (friends, neighbors) and family pet.^19^

Dog bites are commonly associated with soft-tissue injury to the face, but rarely result in facial fractures.^1,4,19,20^ The injuries to the soft tissues are designated into three categories: Lacerations, punctures, and avulsions (tissue loss). The resulting soft-tissue injuries can vary considerably in relation to their extent and depth.^20^ The actual incidence of facial fractures relating to dog attacks is currently unknown. Schalamon et al.,^1^ Karlson,^3^ and Palmer and Rees^16^ documented no maxillofacial fractures in their review of facial dog bite injuries, and Tu et al^20^ suggested that facial fractures may occur in less than 5% of dog attack incidents.^1,3,16,20^ When a maxillofacial fracture is encountered, the most frequent bones to be fractured are the orbital, nasal, and maxillary bones, constituting 78% of the documented dog bite facial fractures.^20,21^ The mechanism of injury in cases of maxillofacial fracture is thought to be the consequence of the mandible (or involved bone) being physically held by the dogs jaws, which is capable of delivering immense force to the area of bone contacted by the dog’s teeth. In some breeds of dog, the force produced has been measured to be in the region of 31,790 kPa.^6,22,23^ The resultant force generated creates a crush-type injury and fracture of the alveolar bone. Young children are especially vulnerable to this type of crush injury, since the maxillofacial skeleton is not completely mineralized, is thinner, and, therefore, considerably weaker compared with during adulthood.^20^ Additional injuries due to animal bite included facial nerve damage, lacrimal duct damage requiring stenting and reconstruction, ptosis from levator transection, and blood loss requiring transfusion.^19^

The severity of the wounds was assessed by Lack-mann’s classification^9^:

I. Superficial injury without involvement of muscle.

II. Deep injury with involvement of muscle.

III. Deep injury with involvement of muscle and tissue defect.

IVa. Stage III in combination with vascular or nerve injury.

IVb. Stage III in combination with bony involvement or organ defect.

The optimal management of these wounds is controversial. The management of dog bite injuries has evolved over the years. In the past, accepted surgical practice involved delayed closure or healing by secondary intention. It was thought that because of the risk of infection, dog bite msinjuries should not be closed primarily.^9,24^ Pinsolle et al^25^ reviewed their series of dog bite injuries between 1979 and 1980. Treatment involved initial surgical exploration, followed by daily dressing with hydrogen peroxide and secondary repair 2 to 7 days later depending on the severity of the injury. More recently, there has been a move to more early and definitive treatment, with authors advocating early washout and debridement of wounds and primary closure.^13,15,26-29^ These changes have arisen from findings that the infection rate increased if treatment was delayed following injury,^30^ that debride-ment reduced the incidence of infection by as much as 30-fold,^30^ and that primary treatment produced the best cosmetic and functional results.^9,10,26,30,31^ Current opinion advocates early surgical treatment with irrigation of the wound, minimal debridement, and direct closure where possible.^9,10,13,16,32,33^ Postoperatively, attention to patient counseling, dressings, ointment, cleaning, and scar revision help assure an optimal outcome for the traumatized tissue. Avulsive injuries with significant tissue loss represent the most difficult cases for definitive management and are also those most likely to require hospitalization.^34^ For traumatic avulsion involving the lip vermilion and the perioral composite soft tissue, even with injuries including delicate anatomic landmarks, healing by secondary intention can be instituted as the initial treatment of choice in younger patients, often providing optimal results.^35^

Our regimen of primary closure after careful debridement of necrotic tissue has been the favored procedure in almost all recent publications.^15,26-29^ Wound cleansing is essential. We irrigated wounds with hydrogen peroxide and saline.^15^ Topical antibiotics and iodine solutions are no longer recommended.^5^ The use of water-based, rather than alcohol-based antiseptic solutions that cannot be used without local anesthesia solutions, is suggested by other authors.^36^

Wound infection is the most common complication following these injuries. Some authors estimate an infection rate of up to 30% following animal bite injuries to the extremities.^37,38^ Most infections caused by mammalian bites are polymicrobial, with mixed aerobic and anaerobic species. Bacteriology of infected dog and cat bite wounds includes Pasteurella multocida, Staphylococcus aureus, Viridans streptococci, Capnocytophaga canimorsus, and oral anaerobes.^19^ Presenting symptoms are usually wound site pain with cellulitis and purulent drainage.^19^ In addition to local wound infection, other complications may occur, including lymphangitis, local abscess, septic arthritis, tenosynovitis, and osteomyelitis. Rare complications include endocarditis, meningitis, brain abscess, and sepsis with disseminated intravascular coagulation, especially in immunocompromised individuals.^19^

Management of infection can be divided into cleansing of the wound, antibiotic prophylaxis, and antibiotic treat-ment.^15^ Antibiotic therapy is indicated for infected bite wounds and fresh wounds considered at-risk for infection, such as extremely large wounds, large hematoma, and cat bites, that appear to be more infected than dog bites (37.5 and 14.9% respectively) and immunocompromised patients.^19^ Antibiotic therapy (a combination of amoxicillin and clavulanic acid) and other combinations of extended-spectrum penicillins with beta-lactamase inhibitors offer the best *in vitro* coverage of the pathogenic flora.^39^ In patients with allergy to penicillins, monotherapy with azithromycin seems to be an effective alternative.^39^ Amoxycillin-clavulanic acid at a dose of 875 + 125 mg, twice a day, by mouth, for adults and 25 mg/ kg, twice a day, by mouth, for children seems to be the best regimen for prophylaxis in bite wound. Alternatively, azithromycin by mouth can be used (for adults 500 mg on day 1 and 250 mg a day for the next 4 days; for infants more than 6 months old, 10 mg/kg on day 1 followed by 5 mg/kg for the next 4 days).^15^ In case of slow recovery or no improvement, simultaneous lymphadenopathy, or pneumonia, *S. aureus* or *Francisella tularensis* should be suspected; ciprofloxacin is recommended.^19^ Prophylactic antibiotics are recommended for 5 to 7 days.^15,40^ Tetanus and rabies prophylaxis must be evaluated in all dog bites.

Metzger et al^36^ proposed the use of antibiotic prophylaxis for patients with comorbidities, high-risk injuries including cat bites, puncture wounds, bites older than 6 hours, extensive trauma to soft tissue, and bites in babies and infants. No antibiotic prophylaxis is necessary for scratch wounds or excoriations.^14,41^ Correira^40^ suggested the use of antibiotic prophylaxis also for patients with an edema at the site of the bite and for patients older than 50 years. Nearly all the patients in the study from Kountakis et al^28^ were given prophylactic antibiotics without regard to the severity of their injuries. Another study that focused on bacteriological background proposed antibiotic prophylaxis after bites by horses and birds.^39^

Prompt assessment and treatment can prevent most bite wound complications.^19^ Early management of such injuries usually guarantees satisfactory outcome. Prevention strategies include close supervision of child-dog interactions, public education about responsible dog ownership and dog bite prevention, stronger animal control laws, better resources for enforcement of these laws, and better reporting of bites.^19^ Anticipatory guidance by pediatric health care providers should attend to dog bite prevention. The need to improve community knowledge of rabies and the availability and affordability of rabies vaccine must be highlighted.^19^
